# The case for FAT10 as a novel target in fatty liver diseases

**DOI:** 10.3389/fphar.2022.972320

**Published:** 2022-11-01

**Authors:** Madushika M. Wimalarathne, Quiana C. Wilkerson-Vidal, Emily C. Hunt, Sharifa T. Love-Rutledge

**Affiliations:** Department of Chemistry, The University of Alabama in Huntsville, Huntsville, AL, United States

**Keywords:** FAT10, fatty liver, non-alcoholic fatty liver, fibrosis, cancer

## Abstract

Human leukocyte antigen F locus adjacent transcript 10 (FAT10) is a ubiquitin-like protein that targets proteins for degradation. TNFα and IFNγ upregulate FAT10, which increases susceptibility to inflammation-driven diseases like nonalcoholic fatty liver disease (NAFLD), non-alcoholic steatohepatitis (NASH), and hepatocellular carcinoma (HCC). It is well established that inflammation contributes to fatty liver disease, but how inflammation contributes to upregulation and what genes are involved is still poorly understood. New evidence shows that FAT10 plays a role in mitophagy, autophagy, insulin signaling, insulin resistance, and inflammation which may be directly associated with fatty liver disease development. This review will summarize the current literature regarding FAT10 role in developing liver diseases and potential therapeutic targets for nonalcoholic/alcoholic fatty liver disease and hepatocellular carcinoma.

## 1 Introduction

### 1.1 The structure and function of F locus adjacent transcript 10

**FIGURE 1:**
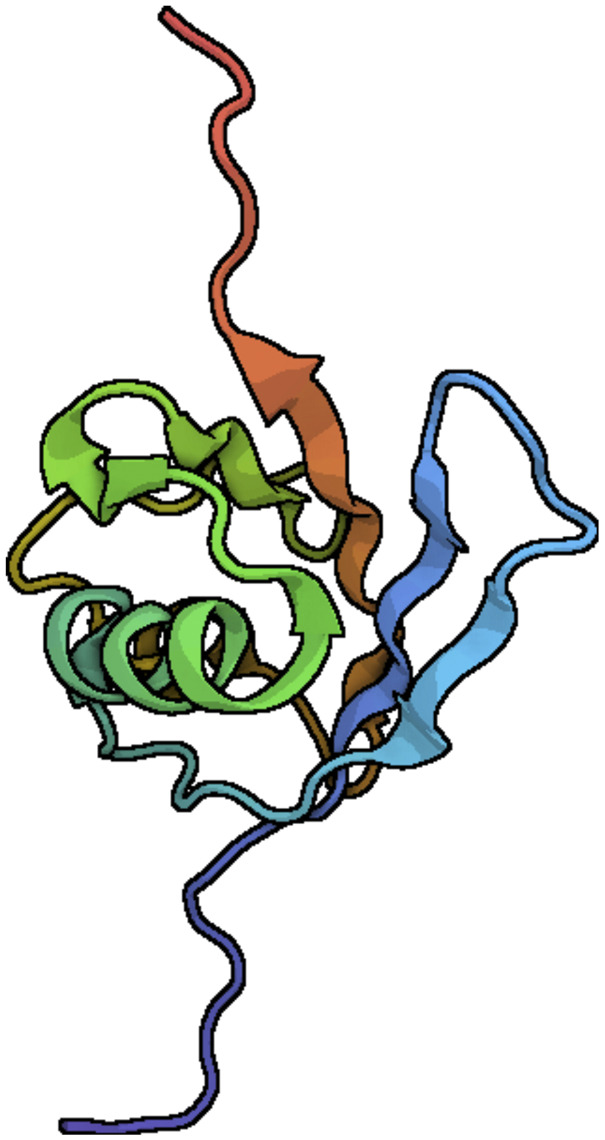
Ribbon diagrams of the FAT10 model structure. N-and C-terminal domains of FAT10 show the typical b-grasp fold. The central α-helix (turquoise) is made with β-sheets (Aichem et al., 2018). The diagrams were generated with the Pymol program, using the Research Collaboratory for Structural Bioinformatics (RCSB) Protein Data Bank entry. PDB ID 6GF2.

Human leukocyte antigen F locus adjacent transcript 10 (FAT10) is a ubiquitin-like protein that is encoded in the major histocompatibility complex (MHC). FAT10 was first discovered in 1996 (Fan, et al., 1996) and located on chromosome six in humans, chromosome 17 in mice, and chromosome 20 in rats (ncbi.nlm.nih.gov). FAT10 is an 18-kDa protein with 165 amino acids, two tandem ubiquitin-like domains, and belongs to the ubiquitin family of proteins (UBL) (uniport.org). The binding domains are 85 amino acids in length, consist of four sheets and three helixes each, and are connected by a flexible linker to form the FAT10 molecule (rscb.org). The FAT10 amino acid sequence is conserved in most mammals except for the C and N terminals (Aichem et al., 2018) The C and N terminals of FAT10 are highly variable even among mammalian species, including *Mus musculus, Rattus norvegicus, Myotis myotis, Mustela erminea, Talpa occidentalis, Gorilla gorilla, Molossus molossus, Mirounga leonina, Hylobates moloch, Nomascus leucogenys* (Figure 1). The clustal omega comparison shows that 60%–70% identity match even among mammalians suggesting FAT10 is rapidly evolving despite being present only in mammals. These evolutionary changes of FAT10 attenuate binding properties and functional characteristics of FAT10 proteins.

FAT10 acts as a modifier to target proteins for degradation. FAT10 is expressed in specific tissues, such as the lymph nodes, kidney, liver, pancreas, and gastrointestinal tract, in response to proinflammatory stimuli. FAT10 is active in several types of cells, including dendritic cells, T cells and B cells (Fan, et al., 1996; Bates et al., 1997) due to cytokine induction of cells (Mah et al., 2020). FAT10 has also been associated with inflammation and the immune response, as part of the MHC, where it plays a role in antigen presentation (Ebstein et al., 2012). It is well known that the inflammatory cytokines tumor necrosis factor *α* (TNF-α), a presumptive tumor promoter, and interferon-γ (IFN-γ) upregulate FAT10 (S. Lukasiak et al., 2008). Furthermore, FAT10 has been shown to interact with the tumor suppressor protein p53 in a mutually inhibitory manner (Zhang et al., 2006; Choi et al., 2014; Zhang and Fu, 2021). Hence, FAT10 has been found to be highly upregulated in cancers of the liver, colon, uterus, and ovaries (Lee et al., 2003; Lukasiak et al., 2008). FAT10 is an important target for research and therapeutics for cancer and inflammation implicating a potential role of FAT10 on inflammation induced tumorigenesis.

FAT10 overexpression is associated with the regulation of several inflammation driving pathways in cancer development, such as the AKT pathway, the Wnt pathway, and the NF-κB (Liu et al., 2014; Yuan et al., 2014; Luo et al., 2018; Zou et al., 2021). In fact, the NF-κB pathway may link inflammation with the development of cancer, making FAT10 important in the mechanism of inflammation-induced tumorigenesis. FAT10 also directly interacts with other downstream targets, such as p53 and β‐catenin (Li et al., 2011; Choi et al., 2014; Yuan et al., 2014). Furthermore, FAT10 has been shown to negatively influence DNA damage repair (Chen et al., 2018). The regulatory role of FAT10 in these pathways makes it important in the progression of cancer.

However, FAT10 is also known to have roles in the development of other diseases, such as kidney disease (Ross et al., 2006) inflammatory bowel disease (Kawamoto et al., 2019) and diabetes (Baschal et al., 2011; Brozzi et al., 2016). The role of FAT10 in the development of inflammation is critical to its role in these diseases and may also contribute to its role in the development of liver diseases. Additionally, FAT10 has been shown to have a role in regulating autophagy and insulin signaling, which may contribute to its role in the development of inflammation and insulin resistance.

## 2 F locus adjacent transcript 10 and liver inflammation

FAT10 expression is known to be increased related to inflammation. FAT10 is presented in the primary insulin dependent diabetes mellitus susceptibility locus (Iddm37) near the major histocompatibility complex II (MHC II) (Fan, et al., 1996) where single-nucleotide polymorphisms in MHC II genes have been linked to an increased risk of drug-induced liver damage (Willemin et al., 2013). The transcript analysis in nonalcoholic fatty liver disease (NAFLD) patients showed that upregulation of proinflammatory cytokines IL-32 and FAT10 levels suggests a possible connection between FAT10 and inflammation responses in liver diseases (Dali-Youcef et al., 2019).

As a result of liver damage and lipid accumulation in the liver, inflammatory cells release cytokines. The significant increase of cytokines, triglycerides and cholesterol adds to the oxidative stress developed in hepatocytes (Yang et al., 2008). The increased mitochondrial damage and ROS production in the liver are associated with fibrogenesis and inflammatory function (Middleton and Vergis, 2021). Hepatic liver inflammation is commonly induced by liver disease by triggering hepatic tissue damage, progressing from NAFLD to severe fibrogenesis and hepatocellular carcinoma (HCC) (Begriche et al., 2013; Buzzetti et al., 2016).

### 2.1 Fat10ylation to major histocompatibility complex class presentation and increased inflammation

Protein homeostasis regulation is a developing topic of research in cancer biology and inflammatory diseases (Calamini and Morimoto, 2012; Cheng et al., 2018). The MHC class I antigen presentation pathway plays a critical role in alerting the immune system (Ebstein et al., 2012). FAT10 serves as a signal for proteasome-dependent degradation and impacts MHC class I presentation. As an example, N-terminal fusion of the human cytomegalovirus (HCMV)-derived pp65 antigen with FAT10 accelerates the proteasomal degradation of pp65 and results in improved presentation in HLA-A2 cells (Ebstein et al., 2012).

All nucleated cells have MHC class I molecules on their cell surfaces, which contain peptide fragments originating from intracellular proteins. In this pathway, covalently attached ubiquitin (Ub) typically marks a substrate protein for degradation by the 26 S proteasome. Furthermore, production of CD8^+^ T cell antigenic peptides mainly depends on the degradation of target proteins by the ubiquitin-proteasome system (UPS) (Lecker et al., 2006). It has been shown that ubiquitylation can facilitate MHC class I antigen presentation (Townsend et al., 1988; Ebstein et al., 2012). In the case of FAT10, substrate protein increases peptide supply for MHC class I-restricted antigen presentation leading to distinctive MHC class I antigen presentation causing changes in inflammation and tumorigenesis (Ebstein et al., 2012). So, we suggest FAT10 modification facilitates MHC class I antigen presentation which may associate with liver inflammation.

A small ubiquitin-like modifier member or SUMO covalently binds to a family of proteins with lysine residues in specific target proteins in a process called SUMOylation. The post-translational protein modification by SUMO is an essential cellular process, involved in protein localization and activation (Seeler and Dejean, 2017). Several isoforms of SUMO have been identified, including SUMO1, SUMO2/3, and SUMO4 (Baczyk et al., 2017; Aichem et al., 2019).

SUMOylation is closely related to the development of liver diseases, including HCC, viral hepatitis, NAFLD, cirrhosis, and primary biliary cirrhosis (PBC) (Zhang et al., 2021).

Alcohol inducible enzyme Cytochrome P450 (CYP2E1) catalyzes reactive oxygen species produced by alcohol (Koop, 2006). The SUMOylation of the pre-oxidant CYP2E1 increases the protein stability and function, resulting in fibrogenesis and inflammation in alcoholic hepatitis patients (Lu and Cederbaum, 2008). The inhibition of SUMOylation in obese mice promotes inflammation by activating the NF-κB pathway, causing liver inflammation (Kim et al., 2015; Zeng et al., 2020). FAT10 binds to SUMO E1-activating enzyme AOS1/UBA2 and competes with SUMO for thioester formation, which reduces the SUMOylation process (Aichem et al., 2019). Furthermore, reduced SUMOylation substantially increases pro-inflammatory immune responses and develops alcoholic steatohepatitis (Decque et al., 2015). So, we suggest that downregulation of SUMO protein activation by FAT10 can induce liver inflammation.

FAT10 and Ubiquitin proteins have different binding specificities which can also affect the rate of proteasomal degradation (Hipp et al., 2005). The two domains of FAT10 are structurally independent and joined by a flexible linker, providing a structural basis for discovering that the two FAT10 ubiquitin domains can dock to different reader domains and thus link two FAT10 binding complexes at the proteasome. For example, this happens with RPN10 and long isoform of NEDD8-ultimate buster 1 (NUB1L) (Groettrup et al., 2008), (Liu et al., 1999). NUB1L is an interferon-induced protein which can bind with NEDD8 and FAT10 and increases FAT10 degradation by around eightfold (Schmidtke et al., 2009). NUB1L has stronger binding affinity toward FAT10 compared to NEDD8 which eventually attenuates the neddylation (covalently conjugating NEDD8 to specific protein), where the neddylation is involved with pathology of NAFLD and NASH (Sen et al., 2015; Yao et al., 2020).

### 2.2 F locus adjacent transcript 10 mediated Mallory Denk Body formation

Mallory Denk Body formation is another example of the impact FAT10-mediated proteasomal degradation processes have in alcoholic hepatitis (AH), alcoholic steatohepatitis (ASH), NASH and HCC (Liu, et al., 2014, Jia et al., 2020). Mallory Denk Bodies (MDBs) are aggresomes formed of undigested ubiquitinated short-lived proteins that have collected due to reducing the 26 S proteasome’s degradation rate. Hepatocyte ballooning and lobular inflammation are required to meet the criteria for MDB formation. 26 S proteasome chymotrypsin activity, measured by the Western blot method in FAT10 KO mice, showed a diminished rate of liver proteolysis compared to wild-type mice, which is associated with MDB formation and balloon degeneration (Oliva et al., 2010). Additionally, betaine prevents FAT10 overexpression in mice fed 3,5-Diethoxycarbonyl-1,4-dihydrocollidine (DCC), preventing MDB formation (French et al., 2012). Consistent with the above data, FAT10 expression increases by 4.5-fold in AH livers compared to NASH and control livers with elevated Mallory Denk Body formation (Jia et al., 2020).

According to Liu H. et al. (2014) an epigenetic mechanism plays a role in MDB formation. SAMe and betaine are methyl donors that prevent the demethylation of histones in the DDC-induced MDB mouse model. The MDB formation is associated with the downregulation of the ufm1 conjugation system (Ufmylation) and FAT10-conjugation system (FAT10ylation) pathways (Jia et al., 2020). As evidenced, MDB formation is associated with liver inflammation and FAT10 induces MDB formation in AH, ASH, and NASH and HCC patients.

### 2.3 F locus adjacent transcript 10 mediated mitochondrial dysfunction induces liver inflammation

**FIGURE 2:**
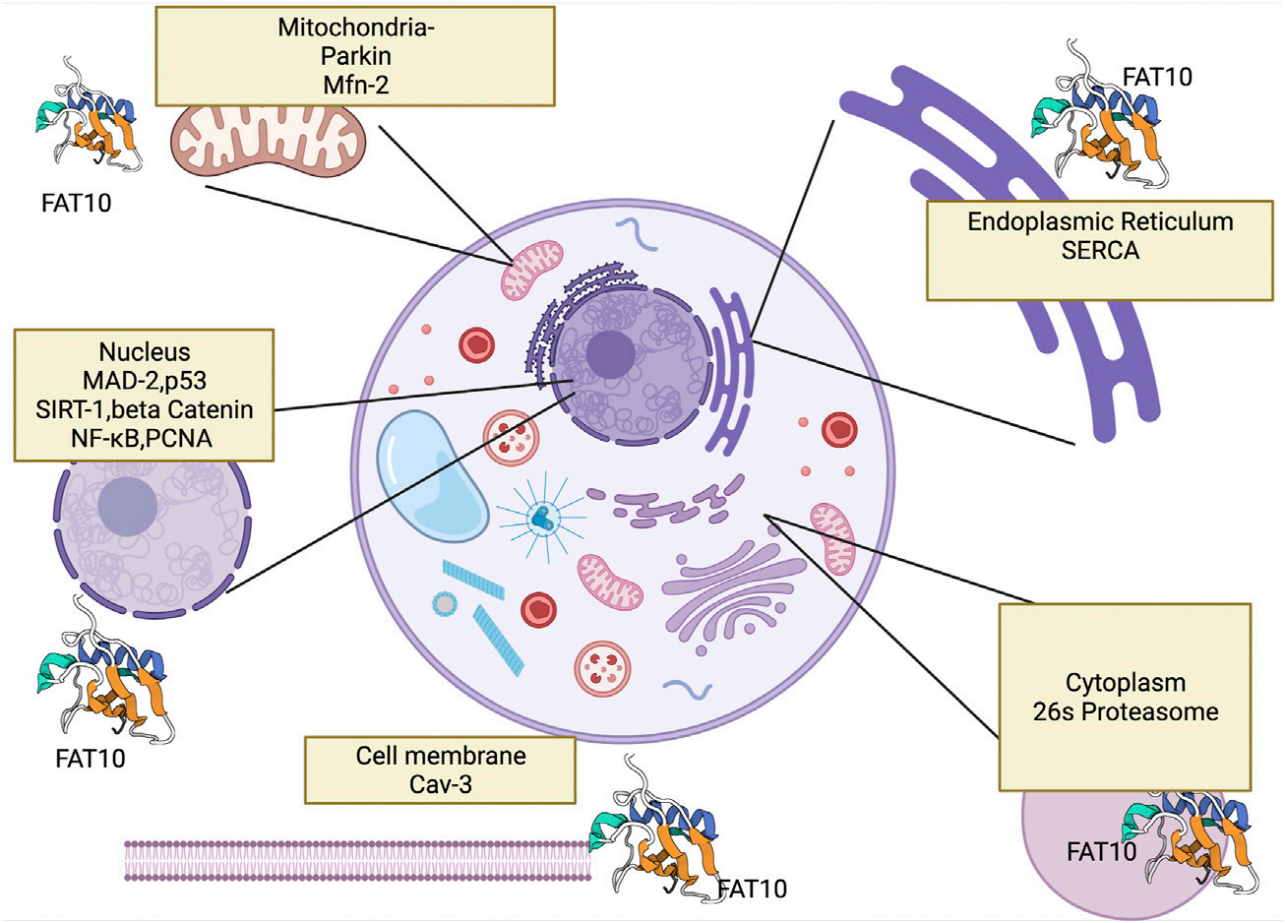
This figure shows FAT10’s interaction with cell organelles and with genes that FAT10 binds. Here we show FAT10 interactions with nucleus (Ramos et al., 2021), (Li et al., 2011) mitochondria (Roverato et al., 2021), cytoplasm (Jia et al., 2020), endoplasm reticulum (Buchsbaum et al., 2012) and cell membrane which may contribute to liver disease development. The figure created by Biorender.com (Accessed on 22nd of March 2021).

The mitochondrion is double-membrane organelle with self-replicating ability. It produces energy through the Krebs cycle, oxidizing fatty acids, and numerous metabolites. For decades, mitochondrial dysfunctions have negatively affected people’s health (Khan et al., 2015). The morphology and replication of mitochondria are mainly regulated by fusion and fission processes (Ni et al., 2015; Zilocchi et al., 2018). Mitochondrial defects are well recognized within human tissue and disease models of alcoholic liver diseases, NAFLD and HCC. Common mitochondrial defects can lead to mitochondrial reactive oxygen species (mtROS) production (Gao et al., 2004) and impaired oxidative phosphorylation, altering hepatocyte cell metabolism, ROS signaling, cell apoptosis, and inflammatory signaling. The mitochondria has several targets of FAT10ylation that affect mitophagy as well as numerous signaling pathways (Figure 2) which have led to changes in inflammation and development of liver diseases (Ren et al., 2011). Increased lipogenesis and elevated free fatty acid (FFA) uptake in hepatocytes are characteristics of the pathogenesis of NAFLD (Petrosillo et al., 2007). Lipotoxicity induces mitochondrial dysfunction, excessive oxidative stress, ER stress, inflammation, and profibrogenic response, predisposing the liver to high-risk conditions. The patients with NASH showed signs of enlarged and swollen hepatocellular mitochondria with a loss of cristae (Middleton and Vergis, 2021), suggesting a close relationship between mitochondrial dysfunction on liver diseases.

Mitofusion or aligning and binding of two mitochondria together to form a larger mitochondrion plays a role in embryonic development, coordination between mitochondria (Chen et al., 2003) and exchange of genetic content between mitochondria. Emerging evidence suggests that mitochondrial fusion responds to chemical and other stresses (Meyer et al., 2017). Upregulated mitochondrial fusion protects against mitochondrial depolarization and promotes cell autophagy in HCC cell lines. The most prominent proteins related to this process are Mitofusion 1 (MFN-1) and Mitofusion 2 (MFN-2), located outside the mitochondrial membrane (Youle and van der Bliek, 2012). MFN-1 and MFN-2 proteins are closely related and play a role in the last steps of mitofusion (Hales and Fuller, 1997). When MFN-2 is responsible for outer membrane fusion, Opa1 protein mediates the fusion of inner membranes (Song et al., 2009; Ge et al., 2018). Mitofission, on the other hand, segregates damaged and malfunctioning mitochondria allowing for degradation *via* the mitophagy process (Middleton and Vergis, 2021). There is no evidence that FAT10 effects the mitofission process.

According to Lehmann et al. (2016) eighty-seven ubiquitin-protein system components, including ubiquitination machinery (E1, E2, and E3 ligases), are localized and interact with 127 mitochondrial matrix proteins in the mitochondria in yeast and humans. Half of these proteins are exclusively located within the mitochondria’s inner matrix, suggesting a strong connection between ubiquitin proteins with mitochondrial regulations. Parkin-dependent fat10lyation of Mitofusion2 was observed in Hek293 cells suggesting that FAT10 proteins present in the outer mitochondrial membrane (OMM) bind to OMM proteins (Roverato et al., 2021). FAT10 mediates the degradation of MFN-2 in neuronal cells, which affects the mitofusion process (Roverato et al., 2021) MFN-2 KO and MFN-1 KO led to severe mitochondrial fragmentation (Papanicolaou et al., 2012; Dong et al., 2016). However, the role of FAT10 in mitochondrial fusion and fission is still to be discovered.

Altered fission and fusion have been observed in tumor cells due to membrane permeability and polarization changes. MFN-1 and MFN-2 activity in the mitochondrial membrane changes with tumor progression and target autophagy, and decreased MFN-2 expression was observed in tumor cells (Boland et al., 2013; Chávez et al., 2017). So, we suggest that FAT10 overexpression may also be associated with reduced MFN-2 levels since FAT10 overexpression can lead to proteasomal degradation of MFN-2.

Mitophagy or selective degradation of mitochondria is another mitochondrial process regulating mitochondrial dynamics. Mitophagy is known for playing a role in paternal mitochondrial degradation (Ma et al., 2020), neurogenerative diseases (Roverato et al., 2021), erythropoiesis (van Vuren et al., 2021) and tissue injuries (Ke, 2020). Impaired mitophagy has been critically linked with the pathogenesis of inflammatory diseases like fatty liver disease and HCC (Ke, 2020; Wu et al., 2020). It was unknown how FAT10 interferes with the mitophagy process until very recently. This connection negatively affects Parkin by changing the structure of the N terminus of the protein, leading to autoFAT10lytion followed by degradation of Parkin (Roverato et al., 2021).

The stress-responsive mitochondrial sirtuin SIRT4 plays a role in the mitochondrial function of NAFLD patients (Tarantino et al., 2014). SIRT4 interacts with GTPase optic atrophy 1 (L-OPA1) to promote mitochondrial fusion, inhibiting mitophagy and increasing ROS production (Lang et al., 2017)**.** Furthermore, TNF-α and IFN-γ induce FAT10 expression, leading to reactive oxygen species (ROS) accumulation and impaired mitophagy (Mah et al., 2020). In summary, FAT10ylation of mitochondrial proteins leads to rapid proteasomal degradation, which eventually alters mitochondrial dynamics like mitofusion and mitophagy leading to fatty liver disease development.

### 2.4 F locus adjacent transcript 10 association with insulin resistance

Decreased insulin sensitivity or insulin resistance is a hepatic component of metabolic syndrome, leading to increased blood glucose, triglyceride, and cholesterol levels. Studies have shown that reduced insulin clearance is associated with increased lipid accumulation in the liver (Seppälä-Lindroos et al., 2002; Utzschneider and Kahn, 2006). Liver insulin resistance is linked to steatosis (Bril et al., 2014), type 2 diabetes (Lukic et al., 2014), NAFLD (Paschos and Paletas, 2009), hepatocellular carcinoma, and cardiovascular disease (Bril et al., 2014).

FAT10 KO mice show increased insulin sensitivity, elevated lipolysis, and insulin-stimulated AKT phosphorylation compared to control rats (Canaan et al., 2014). FAT10 KO mice had upregulated gene and protein expression of fatty acid oxidation in skeletal muscle, and increased lipolysis in adipocytes (Canaan et al., 2014). According to Ge et al. (2018) FAT10 may downregulate insulin receptor substrate 2 (IRS2), decreasing insulin sensitivity in the liver. They hypothesize that the overexpression of FAT10 inhibits the activity of insulin receptor substrates 1 and 2, which leads to the downregulation of PI3K1 expression and causes insulin resistance in mice. The Attie Lab Diabetes database shows relative mRNA expression of FAT10 in the liver of four groups of BTBR mice (4-week-old normal group, 4-week-old obese group, 10-week-old normal group, and 10-week-old obese group, five mice in each group) where ob/ob mice showed increased FAT10 expression compared to lean mice (Kolishovski et al., 2019). BTBR ob/ob mice exhibit insulin resistance and elevated triglyceride levels, suggesting that FAT10 may be associated with metabolic disorders and increased liver inflammation.

These data suggest that FAT10 may play a role in insulin resistance, and the FAT10 pathway may be a practical therapeutic approach to metabolic disorders. However, the relative contribution of FAT10 to insulin resistance is not well established.

## 3 F locus adjacent transcript 10 and liver diseases

Alcoholic liver disease (ALD) and NAFLD have similar pathological characteristics, from simple steatosis to liver cirrhosis, making it difficult to differentiate between them. During the development of fatty liver, increased lipid accumulation and decreased mitochondrial oxidation, liver injury ballooning, changes in lipid composition, and increased ROS production are observed (Ipsen et al., 2018). The fatty accumulation in the liver occurs to a greater degree in NAFLD than in ALD (Toshikuni et al., 2014). On the contrary, inflammation is more pronounced in ALD than in NAFLD (Toshikuni et al., 2014). In addition, venous or perivenular fibrosis, phlebosclerosis, and lymphocytic phlebitis are more common in ALD than in NAFLD (Toshikuni et al., 2014).

FAT10 is known to be associated with the development of liver diseases. The progression of liver disease is described as a “multiple-hit” process (Tilg and Moschen, 2010). After the first hit of insulin resistance, subsequent hits include inflammation, oxidative stress, apoptosis, and fibrogenesis. The development of liver disease may be exacerbated by lifestyle factors, such as alcohol consumption. With each hit, liver disease progresses from fatty liver development to liver disease, steatohepatitis, fibrosis, and hepatocellular carcinoma (HCC). In this section, we will discuss the association of FAT10 with the progression of liver disease. We will follow the progression of liver disease from NAFLD and ALD, NASH and ASH, fibrosis, and finally HCC, discussing how FAT10 is associated with each stage in the development of the disease.

### 3.1 F locus adjacent transcript 10 and alcoholic and nonalcoholic fatty liver diseases

Alcoholic fatty liver pateints showed increased FAT10 expression in the liver along with Mallory Denk body formation (Section 2.3). Alcoholic mediated SUMOylation enhances alcoholic liver disease development *via* upregulating CYP2E1, suggesting FAT10 plays a critical role in alcoholic liver disease development (Section 2.1).

As recently identified, FAT10 plays a role in energy and nutrient sensing, bile acid metabolism, and insulin signaling by modulating pathways like PI3K/AKT/mTOR (Canaan et al., 2014), cAMP-dependent signaling, as well as NF-κB–dependent gene expression (Leng et al., 2014). Some studies have demonstrated that during the development of NAFLD, autophagy is inhibited by the PI3K/AKT signaling pathway *via* both short-term and long-term regulation mechanisms (Mao et al., 2016). AKT is phosphorylated by PI3K and becomes activated, and phospho-AKT can bind and regulate many downstream effectors such as BCL-2 family proteins which play a protective role against NAFLD development (Matsuda et al., 2013). p53, a recognized tumor suppressor protein, acts as a master regulator with pleiotropic effects on metabolism, and is involved in cell apoptosis during NAFLD by regulating the balance between BCL-2 and BAX (Panasiuk et al., 2006). FAT10 has negative effects on p53; downregulating p53 may have a negative downstream effect on BCL-2, leading to increased liver cell death, which suggests possible intervention of FAT10 on NAFLD development.

Another study suggests that FAT10 decreases autophagy through modulating SIRT1 degradation, which increases steatosis, hepatocellular injury, and inflammation in NAFLD. Increased SIRT1 levels in the liver reduce NAFLD development by increasing Nrf2 and HO-1 expression in primary hepatic stellate cells, which are regulated by SIRT1-mediated pathway (Wan et al., 2021). SIRT1 also deacetylates PGC-1α and increases autophagy (Wan et al., 2021) both of which are possibly downregulated by FAT10 overexpression with NAFLD development.

Additionally, NAFLD activity scores (NAS) positively correlate with FAT10 expression, suggesting that FAT10 may contribute to hepatic steatosis and inflammation (Dali-Youcef et al., 2019). Mordes et al. (2005), first mentioned that LEW.1WR1 rats developed fatty liver infiltration. Further research by the group identified increased FAT10 expression in the pancreatic lymph nodes due to a missing short interspersed nuclear element near the promoter region of the gene (Canaan et al., 2014). Recent studies of the livers LEW.1WR1 rats have shown increased glucose intolerance and significant upregulation of FAT10 expression in the livers of the rats (Wilkerson-Vidal et al., 2021). Despite these findings, little is understood about how FAT10 specifically affects the lipid metabolism of the liver.

#### 3.2 F locus adjacent transcript 10, steatohepatitis, and fibrosis

ASH patients have higher FAT10 expression in the liver compared to NASH patients, suggesting FAT10 has an association with liver disease progressions (Jia et al., 2020). Patients also showed increased FAT10 expression which correlate with CXCL9 and CXCL10, supporting solid crosstalk between FAT10 and these proinflammatory cytokines in NASH pathophysiology (Dali-Youcef et al., 2019). Immune cell recruitment is essential to develop NASH from simple steatosis. The chemokine receptor 4 (CXCR4) and 7 (CXCR7) are G-protein-coupled receptors, both significantly upregulated in FAT10-overexpressed NASH liver tissues (Liu et al., 2014). FAT10 was found to activate NF-κB, which in turn upregulates CXCR4/7 in NeHepLxHT and HCT116 cells (Gao et al., 2014). The CXCR4 promotes the recruitment of CD4^+^ T cells in NASH. Furthermore NF-κB-CXCR4/7 pathway induces inflammation by forming Mallory Denk Bodies (MDB) in NASH patients (Wang et al., 2021). Another study with NASH patients shows increased proinflammatory chemokines levels, namely CXCL9, CXCL10, IL-32 in the blood (Dali-Youcef et al., 2019). These findings correlate FAT10 expression plays a role in NASH development. NASH has similar histological features to alcoholic hepatitis (ASH), such as increased lipid accumulation, which may progress to fibrosis, cirrhosis, and ultimately HCC.

*In vitro* experiments have confirmed that FAT10 expression gradually increases in liver fibrosis, cirrhosis, and HCC, suggesting that FAT10 may play a role in fibrosis development (Zhang et al., 2021). Fibrosis is the result of chronic inflammatory reactions, and FAT10 increases inflammation by increasing TNF-α/IFN-γ and NF-κB and STAT3 pathways, all of which were up regulated in fibrosis patients (Jia et al., 2020). Furthermore, NF-κB modulates liver fibrogenesis by regulating hepatocyte injury, inflammatory signals, and fibrogenic responses (Luedde and Schwabe, 2011). Inhibition of NF-κB in Kupffer cells decreases liver fibrosis while activation of NF-κB in hepatocytes and Kupffer cells leads to liver fibrosis. Fibrosis occurs due to chronic liver injury, increased liver inflammation and activated macrophages and myofibroblasts secreting TGFβ and other agonists, which help to release collagens in the liver (Lee and Friedman, 2011). Hepatitis B and C virus infection, alcoholic steatohepatitis, non-alcoholic hepatic steatohepatitis, nonalcoholic fatty liver disease, and hemochromatosis can progress to liver fibrosis with the formation of a fibrous scars in the liver (Phillips et al., 2004; Bataller and Brenner, 2005).

Mitochondrial dysfunction is also observed with fibrosis, and it is known that mitochondria dysfunction contributes to the development and progression of fibrosis. Fibrosis development connects with the innate immune response, and ongoing liver injury leads to activation of inflammation-dependent and independent mechanisms, including secretion of cytokines (Pellicoro et al., 2014) and mitochondrial ROS production from dying hepatocytes (Zhang and Fu, 2021). Mitochondria do not fully function in fibrogenic tissues but actively participate in fibrogenesis. Most importantly, NASH patients with advanced stage fibrosis show increased levels of circulating mtDNA. The mtDNA from hepatocytes release into the bloodstream promoting inflammation through binding to endosomal TLR9 of liver Kupffer cells (Garcia-Martinez et al., 2016). These findings suggest that mitochondrial dysfunction plays a role in fibrosis development in the liver.

Defective mitophagy, such as that resulting from PINK1 deficiency, leads to the accumulation of dysfunctional mitochondria (McLelland et al., 2018). An impairment of mitophagy potentially participates in hepatic fibrosis (Gunton et al., 2003; Mao et al., 2016). The changes in mitophagy increases mitochondrial dysfunction leading to depletion of mtDNA in NASH patients with fibrosis (Schröder et al., 2016). Despite these findings, little is understood about how FAT10 is involved in fibrosis development, but we believe that FAT10 may be a potential therapeutic target in treating patients with NASH-induced liver fibrosis.

#### 3.3 F locus adjacent transcript 10 and hepatocellular carcinoma

Hepatocellular carcinoma is one of the most common organ tumors in the world. HCC is a severe complication of chronic liver diseases, especially advanced liver fibrosis and/or cirrhosis. In carcinoma patients and rodents with liver carcinoma, FAT10 is overexpressed in liver (Lee and Friedman, 2011; Liu et al., 2018). Changes of methylation of the promoter region in the FAT10 gene or binding of key tumorigenic promoters increase the FAT10 expression (Liu et al., 2018).

FAT10 has a strong connection with the inflammatory signaling pathway which eventually leads to HCC development (Liu H et al., 2014). According to (Ren et al., 2011), TNF-α activates the NF-κB pathway, which causes FAT10 gene expression in cells leading to tumorgenesis.

FAT10 also promotes HCC by binding to β-catenin and preventing its degradation, which in turn prevents the degradation of HOXB9. It should be noted that HOXB9 plays a role in tumor metastasis and is regulated *via* the Wnt/β-catenin/TCF4 pathway (Yuan et al., 2014). Notably, cDNA microarray analysis showed that loss of FAT10 inhibits HOXB9 expression in liver carcinoma cells, suggesting FAT10 affects HCC tumor metastasis by regulating HOXB9 (Yuan et al., 2014).

FAT10 is also associated with tumorigenesis *via* its interactions with DNA replication proteins and tumor suppressor proteins. FAT10 has been shown to interact with the damage repair protein proliferating cell nuclear antigen (PCNA). FAT10 and PCNA expression is high in hepatocellular carcinoma tissues; however, the FAT10 expression is reduced in regenerated liver tissues, while PCNA expression is elevated, thus suggesting that the association between FAT10 and PCNA expression is only exhibited in tumor tissues. FAT10 binds with PCNA, leading to proteasomal degradation of PCNA in the nucleus and cytoplasm (Chen et al., 2018), leading to increased tumor cell invasion. FAT10 also interacts with mitotic arrest-deficient 2 (MAD2) and induces tumor malignancy. Abrogation of the FAT10–MAD2 interaction reduces the tumor progression (Theng et al., 2014).

Increased FAT10 inhibits the transcriptional activity of the tumor suppressor p53, a protein that hastens FAT10 protein breakdown. p53 double-negative regulation promotes tumor development in the solid tumor model (Rivlin et al., 2011), suggesting FAT10 has pro-oncogenic function in promoting carcinoma (Aichem and Groettrup, 2016; Dai et al., 2016).

### 4 Conclusion

**FIGURE 3:**
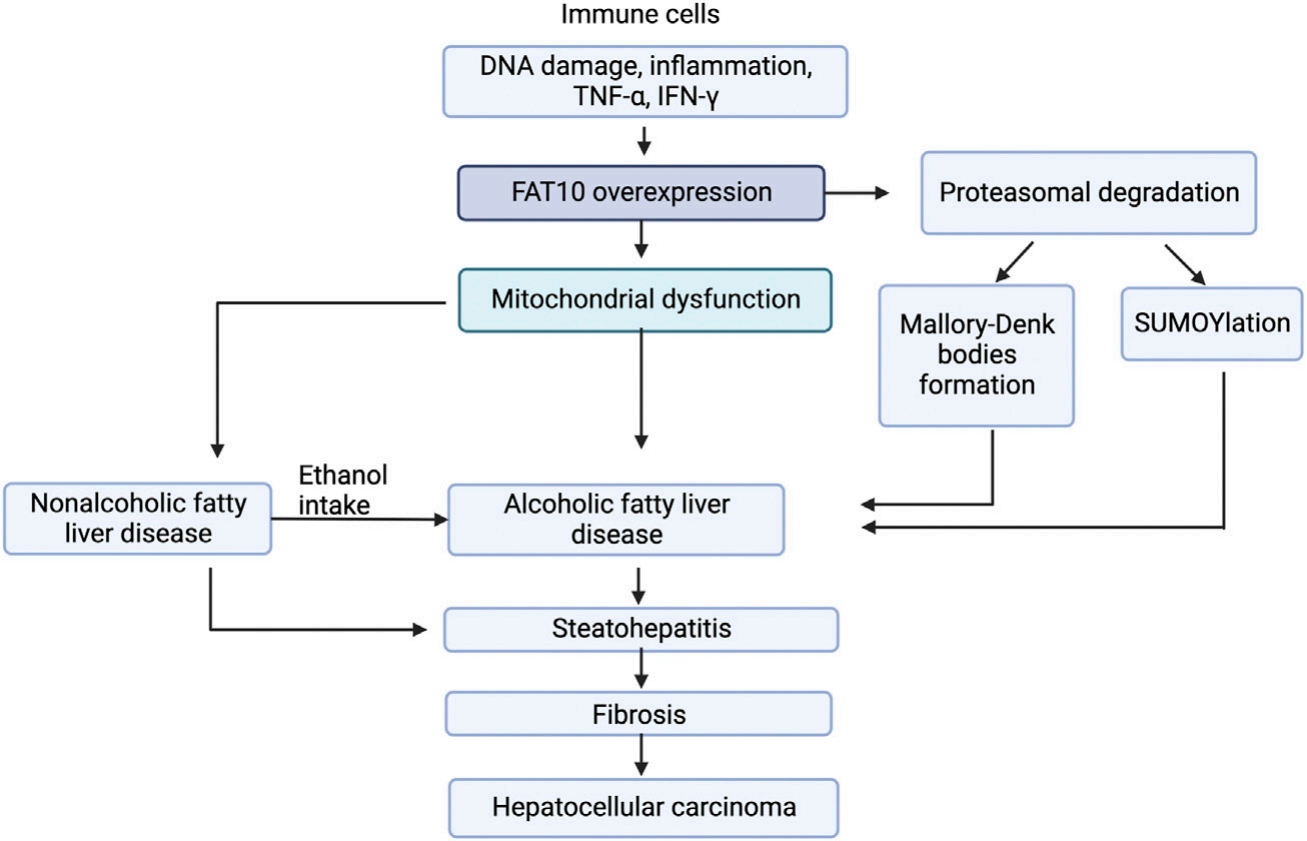
Model for FAT10 effect on liver diseases. The figure shows that FAT10 effects fatty liver diseases *via* altering insulin resistance, mitochondrial function, and inflammation responses. Insulin resistance and mitochondrial dysfunction are closely related to inflammation suggesting potential significance of FAT10 in fatty liver disease development.

These data strongly suggest FAT10 can be used as a prognostic marker for fatty liver diseases and that it is a potential therapeutic target. FAT10 plays a role in prosurvival pathways by altering apoptotic pathways. FAT10 is upregulated in NAFLD, ASH, NASH, and HCC patients (Figure 3). FAT10 may exert its influence in a tissue-specific and cell signaling-specific manner modulating *via* anti-inflammatory mediators, mitochondrial functions, SUMOylation, and MDB formation. The most prominent pathways affected by FAT10 include inflammation, insulin signaling, cell proliferation, mitochondrial protein degradation, and proteasomal degradation.

Research into how FAT10 affects diabetes mellitus and NAFLD could further demonstrate FAT10’s pleiotropic effects in metabolic disorders, but our knowledge of FAT10 and liver diseases is still limited. FAT10 is a biomarker for certain cancers, but it may also serve as a harbinger for the early detection of liver diseases.
